# IL-33 drives airway hyper-responsiveness through IL-13-mediated mast cell: airway smooth muscle crosstalk

**DOI:** 10.1111/all.12593

**Published:** 2015-03-16

**Authors:** D Kaur, E Gomez, C Doe, R Berair, L Woodman, R Saunders, F Hollins, FR Rose, Y Amrani, R May, J Kearley, A Humbles, ES Cohen, CE Brightling

**Affiliations:** 1 Institute for Lung Health, Department of Infection, Immunity and Inflammation, University of LeicesterLeicester, UK; 2 Division of Drug Delivery and Tissue Engineering, Centre for Biomolecular Sciences, School of PharmacyUniversity of Nottingham, UK; 3 MedImmune LtdCambridge, UK; 4 MedImmune PLCGaithersburg, MD, USA

**Keywords:** ASM, IL-33, mast cells

## Abstract

**Background:**

Mast cell localization within the airway smooth muscle (ASM)-bundle plays an important role in the development of airway hyper-responsiveness (AHR). Genomewide association studies implicate the ‘alarmin’ IL-33 in asthma, but its role in mast cell–ASM interactions is unknown.

**Objectives:**

We examined the expression and functional role of IL-33 in bronchial biopsies of patients with and without asthma, *ex vivo* ASM, mast cells, cocultured cells and in a mouse model system.

**Methods:**

IL-33 protein expression was assessed in human bronchial tissue from 9 healthy controls, and 18 mild-to-moderate and 12 severe asthmatic patients by immunohistochemistry. IL-33 and ST2 mRNA and protein expression in human-derived ASM, epithelial and mast cells were assessed by qPCR, immunofluorescence and/or flow cytometry and ELISA. Functional assays were used to assess calcium signalling, wound repair, proliferation, apoptosis and contraction. AHR and inflammation were assessed in a mouse model.

**Results:**

Bronchial epithelium and ASM expressed IL-33 with the latter in asthma correlating with AHR. ASM and mast cells expressed intracellular IL-33 and ST2. IL-33 stimulated mast cell IL-13 and histamine secretion independent of FcεR1 cross-linking and directly promoted ASM wound repair. Coculture of mast cells with ASM activated by IL-33 increased agonist-induced ASM contraction, and *in vivo* IL-33 induced AHR in a mouse cytokine installation model; both effects were IL-13 dependent.

**Conclusion:**

IL-33 directly promotes mast cell activation and ASM wound repair but indirectly promotes ASM contraction via upregulation of mast cell-derived IL-13. This suggests that IL-33 may present an important target to modulate mast cell–ASM crosstalk in asthma.

Asthma is a chronic inflammatory disorder of the airways characterized by inflammation, variable airflow obstruction and AHR. It is estimated that asthma affects 5–10% of adults (1). Genomewide association studies have consistently implicated IL-33 as an asthma susceptibility gene (2–4). IL-33 and its receptor ST2 function as an alarmin to alert the immune system after endothelial or epithelial cell damage during trauma, physicochemical stress or infection (5). IL-33 increased AHR *in vivo* (6) and in lung slices (7) and plays a key role in rhinovirus-induced asthma exacerbations (8). Additionally, disruption of IL-33/ST2 signalling *in vivo* during the course of experimental asthma or anaphylaxis reduced the severity of disease (9–16).

In asthma, mast cell–ASM interactions are important in the development of disordered airway physiology (17). ASM cells from asthmatics express elevated levels of IL-33 compared to healthy subjects (18), and mast cells respond to IL-33 activation (19,20). We hypothesize that the IL-33/ST2 axis plays a role in mast cell–ASM interactions in asthma. We show that IL-33 expression was increased in the bronchial epithelium and ASM in asthma. IL-33 promoted ASM wound repair directly and, in an autocrine manner, augmented mast cell mediator release and, indirectly, increased ASM contraction following coculture with mast cells via upregulation of mast cell-derived IL-13. Similarly, in an *in vivo* mouse model of intratracheal cytokine installation, IL-33 induced AHR which was IL-13 dependent. Therefore, IL-33 may present an important target to modulate mast cell–ASM crosstalk in asthma.

## Methods

A more detailed methods section is provided in the supplement.

### Subjects

Asthmatic subjects had a consistent history and evidence of asthma. The study was approved by Leicestershire Ethics Committee. All patients gave their written informed consent.

### Cell culture

Primary human ASM cells, human lung mast cells (HLMC), human epithelial cells and the human mastocytoma cell-1 (HMC-1) cell line were isolated and cultured as previously described (21–23).

### Animals

Lungs were taken from BALBc (8- to 12-week-old) and C57BL6 (16- to 24-week-old) mice for precision cut lung slicing (PCLS).

### Immunohistochemistry

Bronchial biopsy sections were stained for IL-33 and assessed using a semi-quantitative intensity score (SQS) and quantitative thresholding.

### Flow cytometry and immunofluorescence

IL-33 and ST2 expression was assessed by flow cytometry and immunofluorescence. Cells were counterstained with 4′,6′-diamidino-2-phenylindole (DAPI).

### qPCR

Quantitative RT-PCR of ST2L, ST2 and IL-13 was performed and compared against the internal reference gene 18S.

### ELISA

IL-33 and IL-13 concentrations were quantified by ELISA.

### Calcium flux

The ratio of fluo-3/fura red within cells *vs* time was measured by flow cytometry. Following baseline measurements (1 min), cell flow was halted, IL-33 or calcium ionophore added, and data acquired for a further 3 min.

### Cell metabolic activity assay and apoptosis measurement

ASM cells were treated as indicated in Fig. S1. The CellTiter 96 Aqueous One Solution was added as per the manufacturer's instructions. Apoptosis was assessed by DAPI staining of nuclear morphology and annexin-V ± propidium iodide staining according to manufacturer's protocol.

### Cell contraction

ASM cells ± HLMC (4:1 ratio) were impregnated into collagen gels. Gel surface area was measured using ImageJ (http://rsb.info.nih.gov/ij).

### Mesoscale analysis

Cytokines and chemokines were measured in cells ± IL-33 by electrochemiluminescence detection (Mesoscale Discovery, Gaithersburg, Maryland).

### Wound repair

ASM cells ± IL-33, isotype control or anti-IL-33-neutralizing antibody were wounded as described previously (21). Wounds were photographed at baseline and after 18 h. Wound repair was analysed using cell^F^ software.

### Histamine assay

Histamine was measured in supernatants from activated HLMC (anti-FcεR1 antibody or IL-33 for 24 h) or HLMC incubated with ASM (1:4 ratio) for 5–11 days ± IL-33, isotype control or anti-IL-33-neutralizing antibody, as previously described (24).

### PCLS

PCLS were prepared as described previously (25). Images were captured at baseline, then every 5 min for cumulative carbachol dose responses and 2–10 min post mouse IL-33. Airway lumen size was measured using ImageJ software.

### AHR and inflammation by IL-33

BALBc mice were dosed intranasally with three repeated doses of murine IL-33 (5 μg). Post 3 days, cell number in the lung tissue was assessed by lung digest. AHR was measured using a flexiVent system ± neutralization of IL-13 activity using fusion protein (IL-13Rα2) administered 2 h prior to each IL-33 administration. IL-13, Gob-5 and Muc5AC mRNA expression was determined by RT-PCR, and mouse serum mMCP-1 in serum by ELISA.

### Statistical analysis

Statistical analysis was performed using GraphPad PRISM using parametric and nonparametric tests as appropriate. A *P* < 0.05 was considered significant.

## Results

### IL-33 expression in the ASM-bundle in asthma

**Figure 1:**
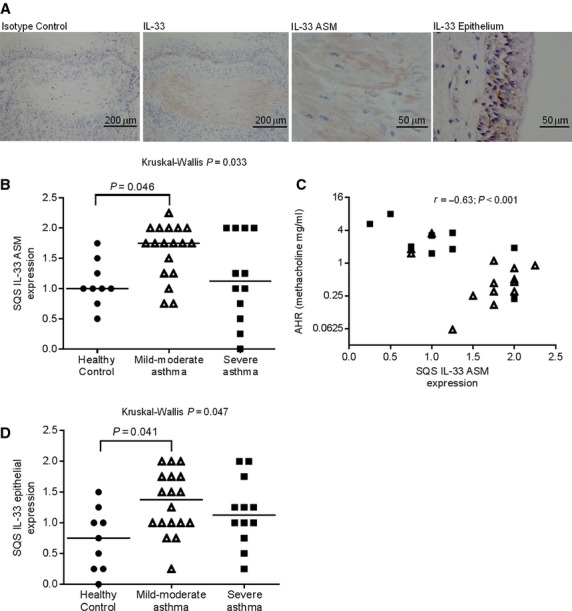
ASM and bronchial epithelial IL-33 expression (A) Representative photomicrographs of bronchial biopsies from an asthmatic subject illustrating isotype control, IL-33+ cell staining in ASM, submucosa (original magnification ×100), and within the ASM-bundle and epithelium (original magnification ×400). (B) Semi-quantitative scoring (SQS) of ASM IL-33. (C) Correlation between SQS ASM IL-33 and AHR in subjects with asthma. (D) Semi-quantitative scoring (SQS) of bronchial epithelium IL-33. • = healthy control, ▵ = mild–moderate asthma, ▪ = severe asthma. Horizontal bars represent the median, *P* < 0.05, Kruskal–Wallis test, for all across-group comparisons.

IL-33 expression was identified within the ASM-bundles in most subjects with variable intensity of expression, and within the epithelium (Fig.1A). Mast cells within the ASM-bundle infrequently coexpressed IL-33 (data not shown). The semi-quantitative intensity score (SQS) for IL-33 expression was significantly increased in mild–moderate asthma compared to healthy controls (Kruskal–Wallis *P* = 0.033; post hoc Dunn's pairwise comparison *P* = 0.046, Fig.1B). The correlation between SQS IL-33 ASM expression and AHR was good (*r* = −0.63, *P* < 0.001, Fig.1C). There was no significant correlation between IL-33 expression and FEV_1_% predicted, bronchodilator reversibility, atopic status or sputum cell counts (data not shown). Epithelial IL-33 expression was also significantly increased in mild-to-moderate asthma compared to healthy controls (Kruskal–Wallis *P* = 0.047; post hoc Dunn's pairwise comparison *P* = 0.041, Fig.1D). The SQS and quantitative expression using thresholding were correlated for both ASM (*r* = 0.63, *P* = 0.004) and epithelium (*r* = 0.43, *P* = 0.013). Quantitative IL-33 expression was increased in the ASM and epithelium in asthmatics compared to healthy subjects, but did not reach statistical significance. Quantitative IL-33 expression in ASM correlated with AHR in those with asthma (*r* = −0.52, *P* = 0.007). The clinical characteristics of the subjects are shown in Table 1.

**Table 1:** Clinical characteristics of asthmatic subjects and healthy controls

	Normal	Mild–moderate asthma GINA 1–3	Severe asthma GINA 4–5
Number	9	18	12
Age†	45 (30–55)	56 (28–63)	51 (45–62)
Male/Female	6/3	5/13	6/6
Atopy *n* (%)	4 (44)	11 (61)	10 (83)
Inhaled corticosteroids BDP μg/day†	0	0 (0–500)	1800 (1600–2000)
Oral corticosteroid mg/day†	0	0	0 (0–7.5)
Methacholine PC_20_FEV_1_ (mg/ml)‡	>16	0.5 (0.3–1.0)*	2.4 (1.2–5.0)*
FEV1% predicted§	95 (3)	86 (5)	77 (7)*
Pre-BD FEV1/FVC %§	82 (2)	72 (2)*	67 (4)*
BD response (%)§	1 (1)	11 (4)*	9 (2)*
Sputum cell counts			
Total cell count (×10^6^/g sputum)†	1.0 (0.8–3.7)	2.5 (0.7–3.2)	3.7 (2.3–7.9)*
Eosinophil %†	0.6 (0.1–0.9)	0.4 (0.1–4.2)	4.0 (2.3–7.9)*
Neutrophil %†	60 (47–71)	51 (25–74)	71 (57–89)
Bronchial biopsy IL-33 expression			
Airway smooth muscle (SQS)†	1 (0.5)	1.75 (0.75)*	1.13 (1.4)
Bronchial epithelium (SQS)†	2 (1)	4 (1.5)*	4 (1.75)

SQS, semi-quantitative score, BDP, beclomethasone dipropionate equivalent.

* *P* < 0.05 compared to control.

† Median (IQR), BD-bronchodilator.

‡ Geometric mean (95% CI).

§ Mean (SEM).

### IL-33 expression by ASM, mast cells and bronchial epithelium

**Figure 2:**
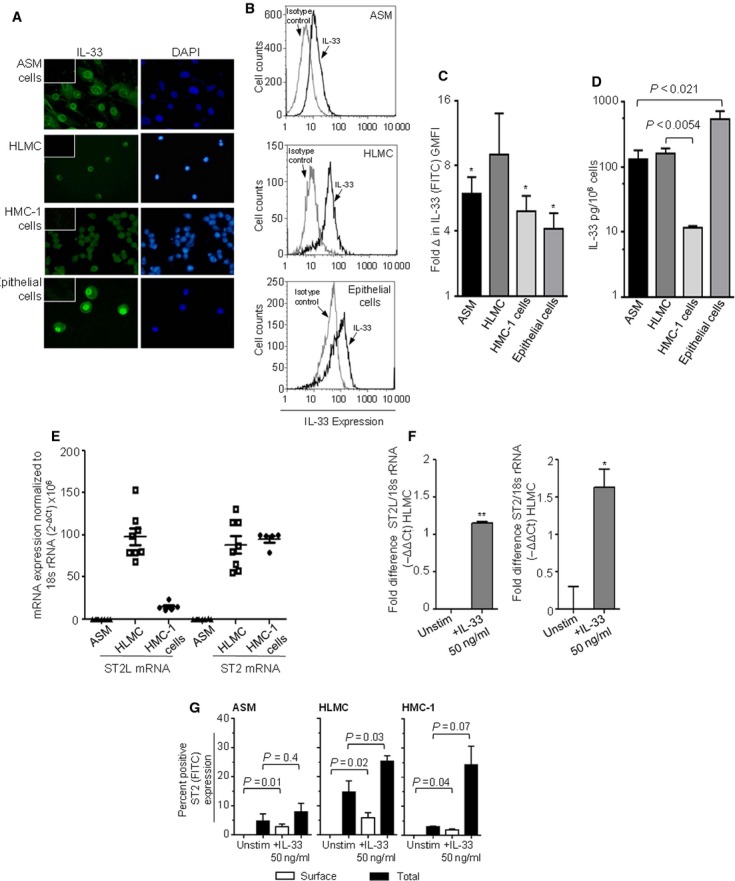
IL-33 and ST2 expressed by *ex vivo* human cells. (A) IL-33 expression was confirmed in ASM, HLMC, HMC-1 and epithelial cells by immunofluorescence (nuclei stained blue, IL-33 stained green, isotype control shown as inset, magnification ×400, *n* = 3). (B) Example flow cytometry histograms in ASM cells, HLMC and epithelial cells represent populations of IL-33 (black line) plotted with the corresponding isotype control (grey line). (C) Quantification of total cell IL-33 in ASM (*n* = 6), HLMC (*n* = 7), HMC-1 (*n* = 5) and epithelial cells (*n* = 11, *<0.05 *vs* isotype control). (D) IL-33 was measured in ASM (*n* = 10), HLMC (*n* = 7), HMC-1 (*n* = 4) and epithelial cell (*n* = 7) supernatants by ELISA. (E) IL-33 receptor (ST2L and ST2) mRNA expression analysed by qPCR in ASM, HLMC and HMC-1 cells. Data were normalized to 18sRNA and expressed following the equation (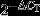)x10^6^). (F) IL-33 receptor mRNA expression in unstimulated and stimulated (IL-33, 50 ng/ml, 24 h) HLMC analysed by qPCR. Data are presented as fold difference on a log_2_ scale (−ΔΔ*C*_t_, *n* = 3,*<0.05, **<0.01 *vs* unstimulated cells). (G) Cell surface and total cell ST2 receptor expression was measured by flow cytometry in unstimulated and stimulated (50 ng/ml, IL-33 for 48 h) ASM, HLMC and HMC-1 cells (*n* = 3). All data presented as mean ± SEM. Statistical differences were assessed using *t*-tests.

IL-33 expression was identified in human ASM, HLMC, HMC-1 and epithelial cells by immunofluorescence (Fig.2A) and flow cytometry (Fig.2B,C). IL-33 expression was not different between ASM cells derived from asthmatic subjects compared to healthy controls (data not shown). IL-33 was spontaneously released from ASM, HLMC, HMC-1 and epithelial cells as measured by ELISA after 24 h (Fig.2D).

mRNA expression of IL-33 receptors ST2L (long transducing isoform) and ST2 (short decoy soluble form) was evident in mast cells, but not in ASM cells (Fig.2E). A two- and threefold increase in ST2L and ST2 mRNA expression were observed respectively in HLMC following IL-33 stimulation (50 ng/ml, 24 h, Fig.2F), but not in ASM cells (*n* = 3, data not shown). Although ST2 cell surface expression was not identified in unstimulated ASM, HLMC and HMC-1 cells by flow cytometry, IL-33 stimulation (50 ng/ml, 48 h) significantly upregulated ST2 surface expression (Fig.2G). Total cell ST2 expression was apparent in all unstimulated cell types and increased poststimulation with IL-33 (Fig.2G).

### Functional responses of ASM and mast cells to IL-33

**Figure 3:**
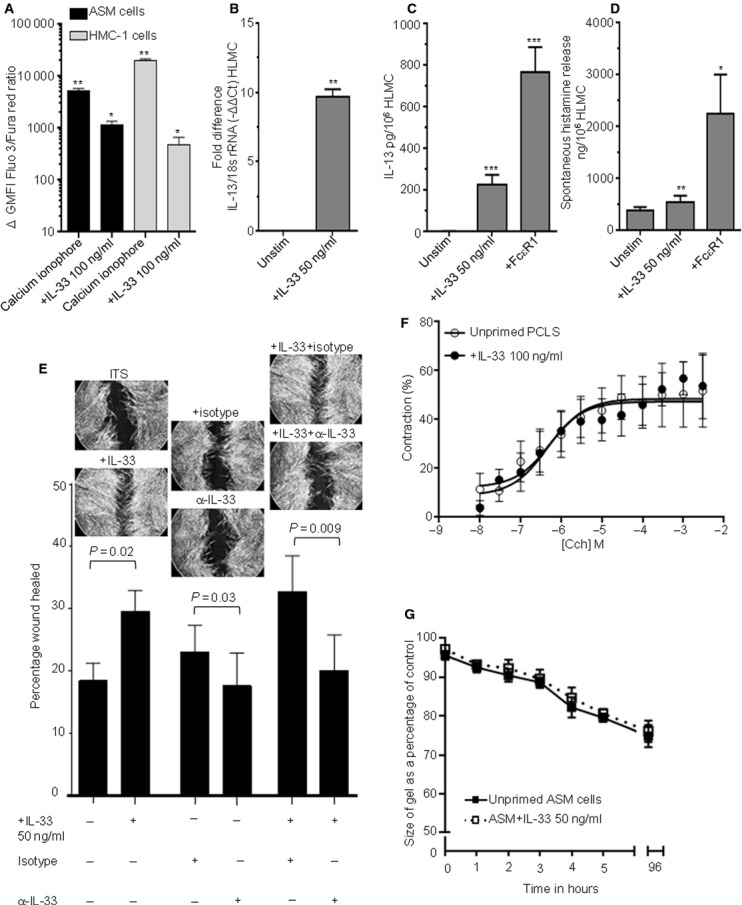
Functional responses to IL-33. (A) Calcium flux in ASM (*n* = 4) and HMC-1 cells (*n* = 4–9) in the presence of IL-33 or calcium ionophore. ΔGMFI equates to total stimulated GMFI minus matched baseline GMFI (*<0.05, **<0.01 compared to baseline GMFI). (B) IL-13 mRNA expression in HLMC ± IL-33 (50 ng/ml, 24 h) analysed by qPCR using the ΔΔ*C*_t_ method. Data are presented as fold difference on a log_2_ scale (−ΔΔ*C*_t_, *n* = 3, **<0.01 *vs* unstimulated cells). (C) IL-13 protein release in HLMC stimulated with anti-FcεR1 (1:1000) and IL-33 (50 ng/ml) for 24 h (*n* = 10–15). (D) Histamine release in HLMC stimulated with anti-FcεR1 (1:1000) and IL-33 (50 ng/ml) for 24 h (*n* = 12–18) (**<0.01, ***<0.001 *vs* unstimulated HLMC). (E) Percentage wound repair by ASM ± IL-33 (50 ng/ml), isotype control or IL-33-neutralizing antibody for 18 h with representative ASM wound repair pictures at 18 h. (F) Percentage contraction in PCLS to cumulative concentrations of carbachol (Cch) pretreated without (unprimed), or with IL-33 (100 ng/ml) for 20 h. Area under the curve gel contraction between group comparisons was made by paired *t*-test. Each point represents mean ± SEM using 1–3 lung slices, 4–8 mice. (G) Collagen gel contraction in ASM cells primed with IL-33 (50 ng/ml over 48 h) impregnated in collagen gels for 3 days (*n* = 4). All data presented as mean ± SEM. Statistical differences were assessed using the t-tests.

IL-33 (50–200 ng/ml) triggered Ca^2+^ flux with increased intracellular calcium in ASM and HMC-1 cells as indicated by an increase in the fluo 3/fura red ratio, with a maximum response at 100 ng/ml (Fig.3A). IL-13 mRNA expression (Fig.3B) and protein release (Fig.3C) were significantly upregulated in HLMC stimulated with IL-33 (50 ng/ml, 24 h) with increased histamine release independent of FcεR1 cross-linking also observed (Fig.3D); HMC-1 (*n* = 4), but not ASM (*n* = 6), cells released CCL2, 4, 5, CXCL8 and 10 significantly following IL-33 incubation (10 ng/ml, 24 h) compared to control (data not shown).

ASM wound repair was promoted by both exogenous and ASM-derived IL-33 (Fig.3E) as demonstrated by an IL-33-neutralizing antibody reducing wound repair in both control and IL-33-treated cells (Fig.3E). Neither ASM proliferation nor survival was modulated by exogenous IL-33 or neutralization of ASM-derived IL-33 (see Fig. S1).

Direct addition of IL-33 (100 ng/ml) to PCLS from BALBc and C57BL6 mice (*n* = 5) did not affect bronchoconstriction over 2–10 min (data not shown). Pretreatment of PCLS from BALBc (Fig.3F) and C57BL6 (*n* = 4, data not shown) mice with IL-33 (100 ng/ml, 20 h) had no effect on Cch-induced luminal diameter. Contraction of human ASM cells primed with IL-33 (50 ng/ml, 48 h) and embedded within collagen gels was not different to unprimed ASM cells over 3 days (Fig.3G).

Previously, we have shown that HLMC/ASM cell coculture promotes HLMC survival/proliferation and results in increased α-SMA expression (23) and histamine release (24). The contribution of endogenous IL-33 to these changes was assessed.

**Figure 4:**
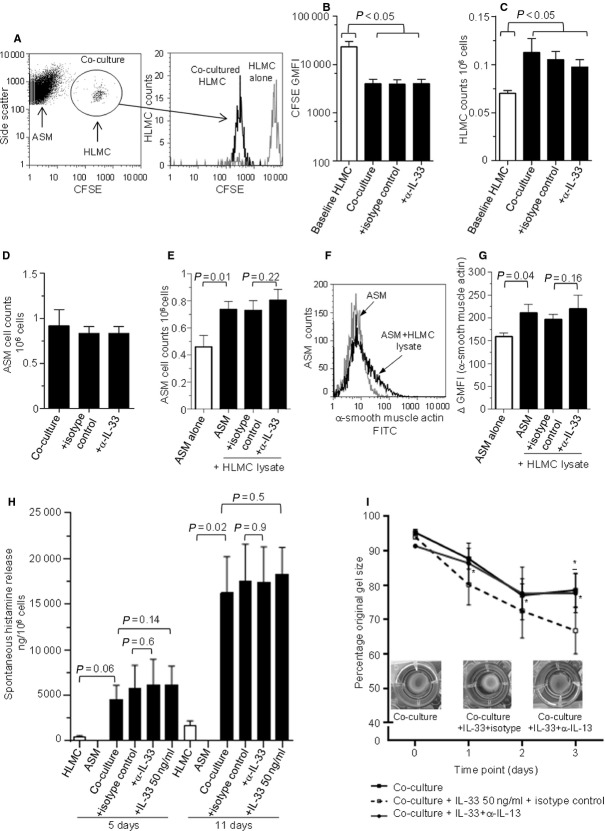
Mast cell and lysate coculture with ASM cells. (A–G) ASM cells were cultured alone or in coculture with HLMC or HLMC lysates and incubated with IL-33 and/or IL-33-neutralizing or isotype control antibodies for 7 days. (A) Representative flow cytometric dot plot showing ASM and HLMC (prelabelled with CFSE) cocultured for 7 days. CFSE-positive cells were gated; histogram shows CFSE GMFI in cocultured HLMC *vs* HLMC alone at baseline. Quantification of HLMC proliferation in (B) indicated by a reduction in CFSE gMFI in ASM/HLMC cocultures (*n* = 4) and (C) by cell counts in ASM/HLMC cocultures. ASM cell counts in (D) following ASM/HLMC coculture (*n* = 4) and (E) with HLMC lysate (*n* = 4). (F) Example histogram of α-SMA expression in ASM cells alone and in coculture with HLMC lysate. (G) ΔGMFI of α-SMA expression in ASM ± HLMC lysate (*n* = 4). (H) Histamine release by HLMC in coculture with ASM (*n* = 4). (I) Collagen gel contraction in cocultured cells ± IL-33 (50 ng/ml), isotype control or IL-13-neutralizing antibody over 1–3 days (*P < 0.05, coculture+IL-33+isotype control *vs* coculture+IL-33+anti-IL-13-neutralizing antibody). Representative gel photographs at day 3. All data presented as mean ± SEM. Statistical differences were assessed using paired *t*-tests (*<0.05).

HLMC cocultured with ASM for 7 days demonstrated increased proliferation compared to HLMC monocultures as determined by CFSE fluorescence (Fig.4B) and cell counts (Fig.4C and D). This was unaffected by IL-33-neutralizing antibody. ASM cells counts were significantly increased following coculture with HLMC lysate for 7 days compared to monoculture; however, this was unaffected by IL-33-neutralizing antibody (Fig.4E).

ASM cells cocultured with HLMC lysate showed increased α-SMA GMFI compared to ASM monocultures, but this was unaffected by IL-33-neutralizing antibody (Fig.4F,G). Histamine release was increased from HLMC following coculture with ASM compared to HLMC monocultures reaching significance after 11 days; however, this was unaffected by IL-33 or IL-33-neutralizing antibody (Fig.4H).

Critically, when both ASM and HLMCs are impregnated into collagen gels following coculture and then stimulated directly with exogenous IL-33, increased gel contraction is seen compared to untreated cells. This can be inhibited by an IL-13-neutralizing antibody but is unaffected by the corresponding isotype control antibody (Fig.4I). HLMCs alone did not elicit gel contraction, ASM/HLMC cocultures did not increase gel contraction compared to ASM alone in the absence of exogenous IL-33, with no effect of IL-13 neutralization over 3 days on this contraction (data not shown, *P* = 0.38, *n* = 3). HLMC IL-13 release was unaffected by coculture with ASM or incubation with ASM-conditioned media (data not shown). These data suggest IL-33 can augment ASM contractility indirectly via upregulation of HLMC IL-13 release. However, endogenous release of IL-33 by ASM is insufficient to activate HLMC IL-13 release in this system.

### IL-33 induces AHR *in vivo* and is IL-13 dependent

**Figure 5:**
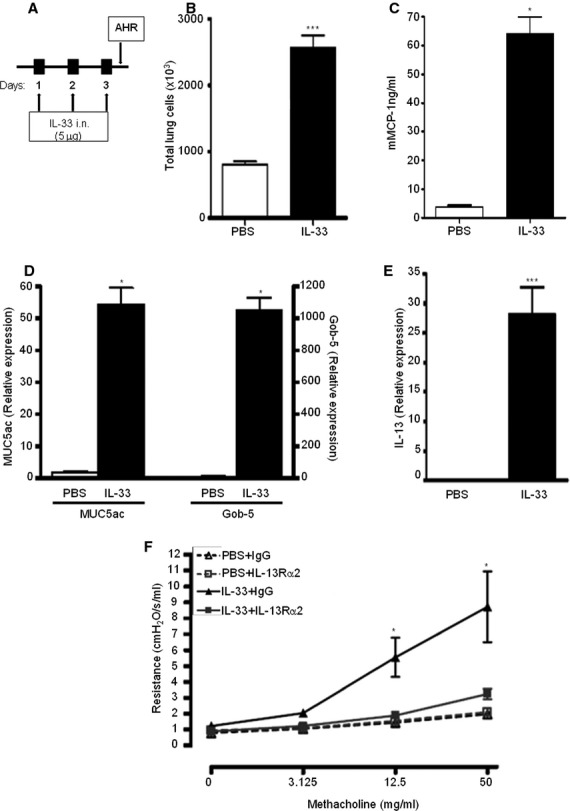
Role of IL-33 in an *in vivo* mouse model. (A) BALBc mice were dosed intranasally with 3 repeated doses of 5 μg (one per day) of murine IL-33. (B) Total cell numbers in lung tissue were assessed after IL-33 administration by lung digest. (C) Mast cell activation was determined by measurement of mMCP-1 release in serum by ELISA. (D) MUC5ac, Gob5 and (E) IL-13 expression assessed in the lung by TaqMan qPCR. All data presented as mean ± SEM, *P < 0.05, ***P < 0.001 compared with PBS control, *n* = 5–18 mice/group from 1-3 separate experiments. (F) IL-13 signalling was blocked using an IL-13Rα2 fusion protein administered 2 h prior to each IL-33 administration. Data are expressed as mean ± SEM, *n* = 5–18 mice/group from 1 to 3 separate experiments, *P < 0.05 by two-way anova compared with PBS control-treated mice.

IL-33 induced a profound AHR in naïve BALBc mice after intranasal challenge with increase in total lung cells (Fig.5B), mast cell activation with increased serum concentrations of mouse mast cell protease-1 (mMCP-1, Fig.5C) and increased expression in the airway of MUC5ac, Gob-5 (Fig.5D) and IL-13 (Fig.5E). Interestingly, similar to results in the human coculture system, neutralization of IL-13 activity (using IL-13Rα2 fusion protein administered 2 h prior to each IL-33 administration) abrogated AHR significantly (Fig.5F).

## Discussion

We demonstrated IL-33 expression *in vivo* and *in vitro* in the bronchial epithelium and ASM and in primary mast cells. The ST2 receptor was expressed by mast cells and ASM by total cell staining and at the surface following IL-33 treatment. IL-33 promoted mast cell activation and ASM wound repair and indirectly promoted contraction via upregulation of mast cell-derived IL-13. This suggests that IL-33 may present an important target to modulate mast cell–ASM crosstalk in asthma.

We report here that IL-33 expression was evident in the bronchial epithelium and ASM-bundle, with expression increased in mild–moderate asthmatics compared to healthy controls. This is consistent with earlier reports in adult asthma (18,26), but contrasts with paediatric severe asthma in which neither epithelial nor ASM IL-33 expression was increased compared to controls (6). We found expression in the ASM, but not epithelium was correlated to the degree of AHR. Primary mast cells and ASM expressed IL-33 and ST2 constitutively as assessed by total cell staining and at the surface following IL-33 treatment. Localization of IL-33 was detected primarily within the nuclei as seen in human nasal fibroblasts (27).

We demonstrated for the first time that IL-33 had no effect on ASM proliferation, apoptosis and synthetic capacity, but both exogenous and ASM-derived IL-33 played an important role in ASM wound repair. Thus, endogenously expressed IL-33 in ASM observed *in vivo* in humans may contribute to ASM repair via migration following damage secondary to physical, mechanical or inflammatory insults. Further work is required to determine the mechanism via which IL-33 stimulates ASM migration; however, in support of our observations, recombinant IL-33 has been shown to have direct effects on chemotaxis of myofibroblasts, fibrocytes, neutrophils, nuocytes and microglia cells (28–31).

IL-33 triggered calcium flux in both ASM and HMC-1 cells, in keeping with other studies showing that IL-33 can enhance calcium elevation autonomously or in synergy with other mediators (32) and that IL-33 can activate calcium-dependent downstream signalling (33–35). Although the mechanism by which IL-33 causes calcium elevation has not been studied, other members of the IL-1 family have been shown to induce calcium signalling in a manner which is GPCR dependent involving both extracellular calcium and intracellular calcium stores (36). Due to the rapid response, the effect of IL-33 on [Ca^2+^]_i_ elevation is likely to be a direct effect on ASM, but in the mast cells it could be a synergistic response in conjunction with preformed mediators released by mast cells.

Importantly, IL-33 is a critical cytokine in the initiation and exacerbation of inflammatory responses and enhanced IgE production in naïve wild-type mice, histamine release (37) and tryptase expression (38) in mouse mast cells. Similarly, human mast cells respond to IL-33 activation (19,20). Here, we found that IL-33 upregulated ST2, IL-13 and histamine release acutely by mast cells independent of FcεR1 cross-linking. Mast cell localization to the ASM-bundle is a notable feature of asthma, and therefore, ASM-derived IL-33 might play an important role in IgE-independent mast cell activation in the asthmatic airway. In addition, mast cell proteases have recently been shown to increase the activity of IL-33 (39).

Indeed, mast cell number within the ASM-bundle is related to the degree of AHR (17). Coculture of primary ASM and mast cells promotes mast cell activation (24), differentiation (40), survival, proliferation and phenotypic changes in ASM (23,24,40). Neutralization of IL-33 in ASM/mast cell cocultures had no effect on mast cell proliferation or histamine release, or α-SMA expression by ASM cells. Interestingly, coculture of ASM and mast cells together with the addition of IL-33 increased collagen gel contraction. This was IL-13 dependent, and the enhanced contraction in response to IL-33 was normalized following IL-13 neutralization. IL-33 had no direct effect on human ASM contraction or *ex vivo* PCLS from BALBc or C57BL6 mice. We found that in a mouse model system, IL-33 induced AHR, mMCP-1, MUC5ac, Gob-5 and IL-13 expression that was abrogated with IL-13Rα2 fusion protein supporting the human findings of IL-13-dependent IL-33 induction of ASM contraction possibly via mast cell activation. These findings are supported by two independent recent studies. Barlow and colleagues (7) examined the response to methacholine in the PCLS *ex vivo* model and demonstrated that IL-33 mediated increased AHR that was IL-13 dependent. Saglani and colleagues (6) found that IL-33 induction of AHR was IL-13 dependent prior to prolonged exposure to house dust mite, but following this exposure was partly IL-13 independent. However, the exact mechanism via which IL-33 mediates IL-13-dependent ASM contraction remains to be elucidated.

Critically, in contrast to human asthma, mast cell localization to the ASM is not a feature of murine models of asthma. Therefore, although the animal models support the concept that IL-33-induced AHR is IL-13 dependent, whether mast cells are critical in these models is uncertain. Indeed, IL-13 has been shown to be produced by Th2 cells (41), NKT cells (42), basophils (43) and ILC2s (44,45), the latter two of which can be dependent on IL-33. Nevertheless, IL-13 release by mast cells in human disease secondary to IL-33 activation remains likely to be important in human disease as these mast cells express IL-13 *in vivo* (46,47) and are the most abundant inflammatory cell in the ASM-bundle (17). Interestingly, we were unable to demonstrate that constitutive ASM-derived IL-33 was sufficient to induce IL-13 release from mast cells in coculture, and therefore, it is likely that either upregulation of IL-33 release by ASM *in vivo* or contributions from other cellular sources such as the epithelium might be important in activating IL-13 release from mast cells in asthma. The exact role of IL-33 in human disease will require future clinical studies targeting the IL-33 axis.

In conclusion, our findings showed that IL-33 promoted mast cell activation and ASM wound repair and indirectly promoted both ASM contraction *in vitro* via upregulation of mast cell-derived IL-13 and also IL-13-dependent AHR *in vivo*. Therefore, IL-33 might be an important novel therapeutic target to modulate mast cell–ASM crosstalk in asthma.

## Supporting Information

**Data S1.** Materials and Methods. **Figure S1**. Role IL-33 ASM cell proliferation and apoptosis.
