# Supplementary material for: IL-33 drives airway hyper-responsiveness through IL-13-mediated mast cell: airway smooth muscle crosstalk
Source: Allergy. 2015 Mar 16;70(5):556–67. doi: 10.1111/all.12593 (PMC4418379; doi:10.1111/all.12593)
Supplement: Supplementary file 1 [file all0070-0556-sd1.doc]

**Online Data Supplement**

**IL-33 drives airway hyper-responsiveness through IL-13 mediated mast cell:airway smooth muscle crosstalk**

Davinder Kaur, Edith Gomez, Camille Doe, Rachid Berair, Lucy Woodman, Ruth Saunders, Fay Hollins, Richard May, Jennifer Kearley, Alison Humbles, Suzanne Cohen, Christopher Brightling.

**MATERIAL AND METHODS**

***Subjects***

Subjects were recruited from Leicester, UK. Asthmatic subjects had a consistent history and evidence of asthma (E1). Asthma severity was defined by Global Initiative for asthma treatment steps (mild-moderate GINA 1-3, severe GINA 4-5 (E2). Subjects underwent extensive clinical characterization including sputum induction (E3) and video-assisted fibreoptic bronchoscopic examination as described previously (E4). The study was approved by the Leicestershire

***Cell isolation and culture***

Pure primary ASM bundles were isolated from bronchoscopic samples (n=31, asthma, n=15, non-asthma n=13) and from lung resection (n=3). ASM was cultured and characterized by immunofluorescence and light microscopy using anti-human alpha smooth muscle actin-FITC conjugated mAb (clone, 1A4) and anti-human myosin mAb (clone SMMS-1, Sigma-Aldrich, Dorset, UK) indirectly conjugated with RPE (Dako, Ely, UK) as previously described (E5). The HLMC were isolated and cultured from non-asthmatic lung (n=42) and lung resection material using anti-CD117-coated immunomagnetic Dynabeads as described previously (E6). The final mast cell purity was 99%. Following purification, HLMC were immediately cultured in DMEM/Glutamax/HEPES media containing antibiotic/antimycotic solution, non-essential amino acids (Invitrogen, Life Technologies, Paisley, UK), 10% FBS (Sigma-Aldrich), and cytokines (100ng/ml SCF, 50ng/ml IL-6, and 10ng/ml IL-10, R&D Systems, Oxfordshire, UK) as described previously (E7). The Human mastocytoma cell line (HMC-1 cells) was a generous gift from Dr. J. Butterfield (Mayo Clinic, Rochester, MN). The cells were maintained in Iscove’s modified DMEM (Sigma-Aldrich, E8). Basal epithelial cells obtained from nasal brushings and bronchoscopy (n=8, asthma, n=8 non-asthma) were grown on collagen (Advanced Bio Matrix, San Diego, California, USA) coated 12-well plates in bronchial epithelial growth medium (BEGM; Lonza, Berkshire, UK) including supplement SingleQuot BulletKit (Lonza), 0.3% Fungizone antimycotic (Invitrogen), and 1% antibiotic-antimycotic. The epithelial cells were expanded onto collagen-coated T75cm^2^ flasks and replenished with fresh medium three times a week (E9).

***Animals***

BALBc mice were purchased from Harlan. The 7-12 week old female mice were used in all experiments. Mice were housed under specific pathogen free conditions. Lungs were taken from 8-12wk-old female BALBc wild type mice and 16-24wk old female C57BL6 wild type mice for PCLS. Before use, mice were kept for a week, under standard conditions in the University of Leicester’s Division of Biomedical Services, with access to water and food. All studies were approved by the University of Leicester.

***Immunohistochemistry***

Sequential 2µm sections were cut from glycomethacrylate embedded bronchial biopsies and stained using an anti-human IL-33 mAb (clone: IL33305B, Enzo life Sciences, Exeter, UK) and appropriate isotype control IgG1 (R&D Systems) respectively (E4). The number of positively stained nucleated cells was enumerated per mm^2^ of the lamina propria and IL-33 expression by the ASM or epithelium was assessed using a semi-quantitative intensity score from no staining=0, low=1, moderate=2 and high=3 staining. Quantitative assessment of IL-33 epithelial and ASM staining was done using a threshholding technique based on the hue, saturation, and intensity (HSI) method for colour detection as described previously (E10). The observer was blinded to clinical characteristics.

***Flow cytometry***

ASM cells were serum starved in insulin/transferrin-sodium selenite (ITS) media (Sigma-Aldrich) for 24h. IL-33 expression was assessed in ASM, HLMC, HMC-1 cells and human epithelial cells using anti-IL-33 mAb (clone IL33305B) by flow cytometry on the BD FACSCanto flow cytometer (BD, Oxford, UK). Cells were stimulated with human recombinant IL-33 (50ng/ml, R&D Systems) for 48h and surface and intracellular ST2 expression detected using goat anti-human ST2/IL-1 R4 biotinylated antibody (R&D Systems), or its appropriate isotype controls goat IgG biotin (Abcam, Cambridge, UK) and indirectly labeled with Streptavidin/FITC (Dako) by 1 colour flow cytometry.

***Immunofluorescence***

Cells were stained using human anti-IL-33 (clone IL33305B) and appropriate isotype control, indirectly labeled with FITC and counterstained with 4’,6’-diamidino-2 phenylindole (DAPI, Sigma-Aldrich). Cells were studied by a standard fluorescent microscope.

***qPCR***

Reverse transcription real time PCR was used to quantify the relative abundance of the two IL-33 receptors isoforms ST2L (the transducing IL1RL1 iso1) and ST2 (decoy receptor IL1RL1 iso2) in both ASM and mast cells. IL-13 was quantified in HLMCs only. Total RNA was isolated from cells using Peq Gold total RNA kit (PeqLab, Ltd, Southampton, UK), followed by DNaseI treatment, according to manufacturer’s instructions. RNA quality and quantity were assessed using a TECAN infinite NANO-QUANT plate reader (Tecan Ltd, Reading, UK) and 1 μg of total RNA from each cell culture was reverse transcribed using SuperScript Vilo cDNA synthesis kit (Invitrogen). Amplification of 1 to 10ng of cDNA per reaction in a final volume of 20μl was performed using the Express SYBR GreenER qPCR SuperMix Universal (Invitrogen) in a Chromo4 Real-Time Detector (Bio-Rad, Herts, UK). After an initial incubation for 2min at 50°C followed by 5min at 95°C, the conditions of amplification were: denaturation at 95°C, annealing at 59°C, extension at 72°C for 36 cycles. A melting curve analysis was performed after each run. All samples were tested in triplicate and 18s rRNA was used for normalization. 18s rRNA was selected as reference gene as it showed a low variability of expression among the different cell types used. Primers used to amplify ST2L, ST2 and IL-13 was designed by PrimerDesign (Southampton, UK). The sequences were as follows: for ST2L (NM_016232), h.ST2L.1465F: GCACTTTGTTCACCAGATTCT and h.ST2L.1551R: CCAGGTAGCATATCTCTCCCA; for ST2 (NM_003856), h.ST2.1338F: TTGTTTGCTGTCTGATCTTTGTAG and h.ST2.1434R: ACCAACGATAGGAGGGAGTG ; for IL13 (NM_002188), h.IL13.128F:TCCCTCTACAGCCCTCAGG and h.IL13.233R:TGTCAGGTTGATGCTCCATACC. The primers used to amplified the reference gene 18s RNA were h.18SRNA.891F: GTTGGTTTTCGGAACTGAGG and h.18SRNA.1090R: GCATCGTTTATGGTCGGAAC (E11). To assess mRNA expression in different cell types, each transcript expression was normalized to the reference gene and determined using the equation 2-ΔCt, where ΔCt = (Ct _target gene_ -Ct _18Sr RNA_, E12) and this value was arbitrary multiplied by the factor 10^6^ for clearer presentation. The ΔΔCt method (E13) was used to compare the expression of each transcript in stimulated versus unstimulated control conditions. Each transcript expression was then normalized to the 18sRNA reference gene and presented relative to a calibrator (unstimulated cells condition). Data were presented as fold difference on a log^2^ scale (-ΔΔCt).

***ELISA***

The concentration of IL-33 within ASM, mast cells and epithelial cell supernatants were quantified using IL-33 ELISA according to manufacturer’s instructions (R&D Systems). The sensitivity limit of IL-33 assay was 11.7 pg/ml. IL-13 was quantified in HLMC supernatants post IL-33 (50ng/ml) and anti-FcεR1 stimulation using the IL-13 ELISA (eBioscience, Hatfield, UK). The sensitivity limit of IL-13 assay was 1.6 pg/ml.

***Flow Cytometric Measurement of Intracellular Ca^2+^*** ***[Ca^2+^]_i_ responses in cultured cells***

Cells were labelled in parallel with 10μg/ml fluo-3 and 4μg/ml fura red acetoxymethyl esters (Invitrogen) for 45min at 37°C in Phosphate Saline solution +2mM Ca^2+^. For determination of cellular response, baseline unstimulated measurements (1min) were followed by addition of recombinant IL-33 (50-200ng/ml) or calcium ionophore (1.5μg/ml). The cell flow was halted during this addition appended and then continued acquiring for a further 3min. Data was collected in histogram displaying the ratio of fluo-3/Fura Red vs. time. The GMFI of the unstimulated cell population was compared against the stimulated population.

***Cell metabolic activity assay***

ASM cells were seeded at 2 x 10^3^ cells/per well in 10% FBS media over 20h at 37ºC replaced with serum free media for 20h to synchronise the cells. Cells were then exposed to 10% FBS media or ITS media with and without recombinant IL-33 (12.5-100ng/ml), neutralizing anti-IL-33, isotype, 1μM Staurosporine (Sigma-Aldrich) or DMSO control over 96h. The CellTiter 96 Aqueous one solution with the tetrazolium compound (3-(4,5-dimethylthiazol-2-yl)-5-(3-carboxymethoxyphenyl)-2-(4-sulfophenyl)-2H-tetrazolium, inner salt: MTS, Promega, Southampton, UK) was then added to each well according to manufacturer’s instructions.

***Morphological detection of ASM apoptosis***

Morphological features of apoptosis (nuclear condensation and fragmentation) were assessed by DAPI staining. ASM were seeded at 2 x 10^4^ cells/well into 8-well chamber slides and incubated at 37ºC for 24-48h. The media was then replaced with serum free media and incubated at 37ºC for 20h. After ASM cell serum deprivation serum free media was removed and replaced with fresh ITS media ± 100ng/ml IL-33, isotype or neutralizing goat anti-human IL-33 (R&D Systems). The ASM cells were cultured over 96h. ASM cells were stained with DAPI alone and mounted. For each ASM 8 random high powered fields (HPF) were examined (x40) for morphologic features of apoptosis such as nuclear condensation and DNA fragmentation.

***Apoptosis***

**The percentage of apoptotic** ASM cells exposed to 5% FBS media ± 100ng/ml of IL-33 was identified by staining with FITC-conjugated Annexin V (1µl/200µl binding buffer, Bioscience) ± propidium iodide (PI, 0.5μg/ml, Bioscience) after 7 days, prior to analysis on  the flow cytometer. Cell counts were acquired at baseline and after 7 day culture.

***Assessment of cell contraction by collagen gel analysis***

A total of 1.25-2.5 x 10^5^ ASM cells were resuspended in 144μl of ITS media, with 299μl collagen, 37μl 10X DMEM (Invitrogen) and 20μl sodium bicarbonate (Invitrogen), and added to 24-well plates. The mixture was left to polymerize into gels at 37˚C for 90min prior to detachment from the well, 500μl of ITS media ± stimulus was added, and incubated over 4 days. For co-cultured cells a 1: HLMC, to 4 ASM ratio was applied and cells were added to the gel mixture as for ASM cells. Gel surface area was measured using ImageJ (<http://rsb.info.nih.gov/ij>).

***Co-culture HLMC:ASM***

HLMC proliferation was assessed using the Cell Trace carboxyfluorescein succinimidyl ester Proliferation Kit (CFSE, Invitrogen). HLMC were labeled with 2.5µM CFSE for 15min in PBS, prior to incubation in 10% FBS media for a further 30min to allow cleavage of the acetate groups to yield highly fluorescent CFSE and then incubated in 10% FBS at 37ºC. Pre-labeled CFSE HLMC were added in ITS media at a 1:4 ratio to ASM cells and cultured over 7 days ± isotype control or neutralizing anti-IL-33. Cell counts of HLMC were established at baseline with kimura/trypan blue and over 7 day cultures with ASM and CFSE fluorescence analyzed on the flow cytometer.

***ASM co-culture with HLMC lysate***

ASM cells were incubated at 37ºC until cells reached 80% confluence prior to incubation with ITS media at 37ºC for 24h. HLMC lysate were added at 1:4 ratio and cultured for 7 days ± isotype control or neutralizing anti-IL-33. After 7 days ASM cells were harvested and stained with FITC conjugated alpha-smooth muscle actin or IgG2a-FITC isotype control and cell counts established by trypan blue.

***Mesoscale analysis of supernatants from ASM and HMC-1 cells***

The concentration of a panel of cytokines and chemokines were measured in ASM and HMC-1 cells unstimulated and stimulated with recombinant IL-33 (10ng or 50ng/ml) for 24h measured by electrochemi-luminescence detection and pattern arrays (Mesoscale Discovery, Gaithersburg, Maryland, USA). The panel included the cytokines (IFN-γ, IL-1β, IL-2, IL-4, IL-5, IL-10, IL-12p70, IL-13, TNF-α) and chemokines (CCL2, 4, 11, 13, 17, 22, 26 and CXCL8, 10). The limits of detection of the mesoscale system were 2.4pg/ml for all cytokines and chemokines.

***Wound repair***

ASM cells were seeded onto 6 well plates coated with 10μg/ml fibronectin at a density of 0.25 x 10^6^ cells and allowed to adhere and reach 90-100% confluence. Cells were then serum deprived in ITS media and wounded using a sterile 200μl pipette tip in a predetermined grid pattern (E5). Following wounding ASM cells were washed x4 with ITS media prior to addition of ITS media ± IL-33 (12.5-100ng/ml) in the presence or absence of isotype control or neutralizing IL-33. Wounds were then photographed at baseline and after 18h. The percentage of cells that had moved into the wound was analysed using cell F software.

***Histamine assay***

HLMC were activated using anti-FcεR1 antibody (1:1000, Millipore, Ltd, Watford, UK) or IL-33 (50ng/ml) for 24h and supernatants collected for histamine release. ASM (80% confluent) were incubated with HLMC (1:4 ratio) for 5-11 days ± IL-33, isotype, neutralizing anti-IL-33 and supernatants collected. Histamine was measured by sensitive radioenzymatic assay based on the conversion of histamine to methylhistamine in the presence of the enzyme histamine-N-methyltransferase as previously described (E6).

***Precision cut lung slices (PCLS)***

PCLS were prepared as described previously (E14). Briefly, mice were sacrificed by dislocation of the neck. The trachea was canulated, and both lungs were filled with low melting point agarose solution 2% (w/v). The inflated lungs were dissected from the thoracic cavity and placed in cold assay buffer. The lung lobes were separated and PCLS (200μm thick) were obtained using the Campden tissue slicer (Loughbrough, UK). The PCLS were placed into 24 well plates in DMEM (10% FBS, antibiotic-antimycotic, Sigma-Aldrich) and incubated at 37°C. To remove the agarose from the airways, the medium was changed every half hour for the first 2h. Suitable airways on slices were selected on the basis of beating cilia. PCLS were placed in 24 well plates in 1ml assay buffer and observed using an inverted microscope (Nikon Eclipse TE200) attached to a camera (Hamamatsu Digital camera). Airways were located (x100 magnification) and initial baseline images captured (via Openlab) following increased concentrations of carbachol (Sigma-Aldrich, 0.01μM-1000μM). Images were captured every 5min for cumulative dose responses and 2, 5, 10 min post mouse recombinant IL-33 (100ng/ml, R&D Systems). Airway lumen size was measured using Image J software. Airway luminal area at baseline before the addition of stimulant was defined as 100%. Bronchial constriction was expressed as the percentage decrease in luminal area compared with the control airway area at baseline.

***Induction of AHR and inflammation by IL-33 administration***

BALBc mice were dosed intranasally with three repeated doses (one per day over three days) of murine recombinant IL-33 (5μg; Axxora). Post three days total cell number in the lung tissue was assessed by lung digest as previously described (E15). Airway hyper-responsiveness was measured using a FlexiVent system (Scireq, AZ, USA) as previously described (E16) ± neutralization of IL-13 activity using fusion protein (IL-13Rα2; 200μg/mouse; R&D Systems) administered 2h prior to each IL-33 administration. IL-13, Gob-5 and Muc5AC mRNA expression was measured after mRNA was purified with an RNAeasy Plus mini kit (Qiagen, California, USA) and cDNA was synthesized using Sprint Power Script Double Pre-primed 96 kit (Clontech, California, USA). Gene expression was determined by TaqMan® real-time PCR (Invitrogen) following the manufacturer’s protocol. Taqman reactions contained either the reference gene GAPDH or the genes of interest (Invitrogen). Mouse serum mMCP-1 was measured in serum by ELISA according to the manufacturer’s protocol (Moredun Scientific, Ltd., Edinburgh, UK).

***Statistical analysis***

Statistical analysis was performed using PRISM Version 4 (La Jolla, California). Parametric data were presented as mean (SEM) and non-parametric data as median (interquartile range [IQR]). Parametric data were analyzed with paired and unpaired t-tests or one-way analysis of variance and Tukey’s post-hoc test for intergroup comparison as appropriate. Non-parametric data were analyzed using Mann-Whitney tests or the Kruskal-Wallis tests and Dunn’s test for post hoc comparison as appropriate. Correlations between parametric data were assessed by Pearsons correlation and non-parametric data by Spearman’s rank correlation. A p value of p<0.05 was considered significant.

**Supplement References**

E1. [Brightling, C.E](http://www.ncbi.nlm.nih.gov/pubmed?term=Brightling CE%5BAuthor%5D&cauthor=true&cauthor_uid=15879427)., A.J. [Ammit](http://www.ncbi.nlm.nih.gov/pubmed?term=Ammit AJ%5BAuthor%5D&cauthor=true&cauthor_uid=15879427), D. [Kaur](http://www.ncbi.nlm.nih.gov/pubmed?term=Kaur D%5BAuthor%5D&cauthor=true&cauthor_uid=15879427), J.L. [Black](http://www.ncbi.nlm.nih.gov/pubmed?term=Black JL%5BAuthor%5D&cauthor=true&cauthor_uid=15879427), A.J. [Wardlaw](http://www.ncbi.nlm.nih.gov/pubmed?term=Wardlaw AJ%5BAuthor%5D&cauthor=true&cauthor_uid=15879427), J.M. [Hughes](http://www.ncbi.nlm.nih.gov/pubmed?term=Hughes JM%5BAuthor%5D&cauthor=true&cauthor_uid=15879427), and P. [Bradding](http://www.ncbi.nlm.nih.gov/pubmed?term=Bradding P%5BAuthor%5D&cauthor=true&cauthor_uid=15879427). The CXCL10/CXCR3 axis mediates human lung mast cell migration to asthmatic airway smooth muscle. [Am J Respir Crit Care Med.](http://www.ncbi.nlm.nih.gov/pubmed/?term=Brightling+CE%2C+Ammit+AJ%2C+Kaur+D%2C+et+al.+The+CXCL10%2FCXCR3+axis+mediates+human+lung+mast+cell+migration+to+asthmatic+airway+smooth+muscle.+2005.+Am.+J.+Respir.+Crit.+Care+Med.+171%3A1103-1108.) 2005;171(10):1103-8.

E2. Global Initiative for Asthma guidelines. Available at: http:// [www.ginasthma.com](http://www.ginasthma.com/) (accessed 9th May 2011).

E3. [Pavord ID](http://www.ncbi.nlm.nih.gov/pubmed?term=Pavord ID%5BAuthor%5D&cauthor=true&cauthor_uid=9227713), Pizzichini MM, [Pizzichini](http://www.ncbi.nlm.nih.gov/pubmed?term=Pizzichini E%5BAuthor%5D&cauthor=true&cauthor_uid=9227713) E and [Hargreave](http://www.ncbi.nlm.nih.gov/pubmed?term=Hargreave FE%5BAuthor%5D&cauthor=true&cauthor_uid=9227713) FE. The use of induced sputum to investigate airway inflammation. Thorax. 1997;52:498–501.

E4. [Brightling CE](http://www.ncbi.nlm.nih.gov/pubmed?term=Brightling CE%5BAuthor%5D&cauthor=true&cauthor_uid=12037149), [Bradding](http://www.ncbi.nlm.nih.gov/pubmed?term=Bradding P%5BAuthor%5D&cauthor=true&cauthor_uid=12037149) P, [Symon](http://www.ncbi.nlm.nih.gov/pubmed?term=Symon FA%5BAuthor%5D&cauthor=true&cauthor_uid=12037149) FA, [Holgate](http://www.ncbi.nlm.nih.gov/pubmed?term=Holgate ST%5BAuthor%5D&cauthor=true&cauthor_uid=12037149) ST, [Wardlaw](http://www.ncbi.nlm.nih.gov/pubmed?term=Wardlaw AJ%5BAuthor%5D&cauthor=true&cauthor_uid=12037149) AJ, and [Pavord](http://www.ncbi.nlm.nih.gov/pubmed?term=Pavord ID%5BAuthor%5D&cauthor=true&cauthor_uid=12037149) ID. Mast-cell infiltration of airway smooth muscle in asthma. [N Engl J Med.](http://www.ncbi.nlm.nih.gov/pubmed/?term=Brightling+CE%2C+Bradding+P%2C+Symon+FA%2C+et+al.+Mast-cell+infiltration+of+airway+smooth+muscle+in+asthma.+2002.+N.+Engl.+J.+Med.+346%3A1699-1705.) 2002;346(22):1699-705.

E5. [Kaur D](http://www.ncbi.nlm.nih.gov/pubmed?term=Kaur D%5BAuthor%5D&cauthor=true&cauthor_uid=16959919), Saunders R, [Berger](http://www.ncbi.nlm.nih.gov/pubmed?term=Berger P%5BAuthor%5D&cauthor=true&cauthor_uid=16959919) P, [Siddiqui](http://www.ncbi.nlm.nih.gov/pubmed?term=Siddiqui S%5BAuthor%5D&cauthor=true&cauthor_uid=16959919) S, [Woodman](http://www.ncbi.nlm.nih.gov/pubmed?term=Woodman L%5BAuthor%5D&cauthor=true&cauthor_uid=16959919) L, [Wardlaw](http://www.ncbi.nlm.nih.gov/pubmed?term=Wardlaw A%5BAuthor%5D&cauthor=true&cauthor_uid=16959919) A, et al. Airway smooth muscle and mast cell-derived CC chemokine ligand 19 mediate airway smooth muscle migration in asthma. Am J Respir Crit Care Med. 2006;174(11):1179-88.

E6. Sanmugalingam D, Wardlaw AJ and Bradding P. Adhesion of human lung mast cells to bronchial epithelium; evidence for a novel carbonhydrate-mediated mechanism. J. Leukocyte Biol. 2000;68:38-46.

E7. Duffy SM, Lawley WJ, Kaur D, Yang W, and Bradding P. Inhibition of human mast cell proliferation and survival by tamoxifen in association with ion channel modulation. J. Allergy Clin. Immunol. 2003;112: 970–977.

E8. Duffy SM, Leyland ML, Conley EC, and Bradding P. Voltage-dependent and calcium-activated ion channels in the human mast cell line HMC-1. J Leukoc Biol 2001;70:233-40.

E9. Woodman L, [Wan](http://www.ncbi.nlm.nih.gov/pubmed?term=Wan WY%5BAuthor%5D&cauthor=true&cauthor_uid=23238614) WY, [Milone](http://www.ncbi.nlm.nih.gov/pubmed?term=Milone R%5BAuthor%5D&cauthor=true&cauthor_uid=23238614) R, [Grace](http://www.ncbi.nlm.nih.gov/pubmed?term=Grace K%5BAuthor%5D&cauthor=true&cauthor_uid=23238614) K, [Sousa](http://www.ncbi.nlm.nih.gov/pubmed?term=Sousa A%5BAuthor%5D&cauthor=true&cauthor_uid=23238614) A, [Williamson](http://www.ncbi.nlm.nih.gov/pubmed?term=Williamson R%5BAuthor%5D&cauthor=true&cauthor_uid=23238614) R, et al. Synthetic response of stimulated respiratory epithelium: modulation by prednisolone and iKK2 inhibition. Chest. 2013;143(6):1656-66.

E10. Woodman L, Siddiqui S, Cruse G, Sutcliffe A, Saunders R, Kaur D, et al. Mast cells promote airway smooth muscle cell differentiation via autocrine up-regulation of TGF-beta 1. J Immunol. 2008;181(7):5001-7.

E11. Sutcliffe A, Hollins F, Gomez E, Saunders R, Doe C, Cooke MS, et al. Increased NOX4 expression mediates intrinsic airway smooth muscle hyper-contractility in asthma. American Journal of Respiratory and Critical Care Medicine. 2012;185(3):267-74.

E12. [Grosso DF](http://www.ncbi.nlm.nih.gov/pubmed?term="Del Grosso F"%5BAuthor%5D), [Coco](http://www.ncbi.nlm.nih.gov/pubmed?term="Coco S"%5BAuthor%5D) S, [Scaruffi](http://www.ncbi.nlm.nih.gov/pubmed?term="Scaruffi P"%5BAuthor%5D) P, [Stigliani](http://www.ncbi.nlm.nih.gov/pubmed?term="Stigliani S"%5BAuthor%5D) S, [Valdora](http://www.ncbi.nlm.nih.gov/pubmed?term="Valdora F"%5BAuthor%5D) F, [Benelli](http://www.ncbi.nlm.nih.gov/pubmed?term="Benelli R"%5BAuthor%5D) R, et al. Role of CXCL13-CXCR5 crosstalk between malignant neuroblastoma cells and Schwannian stromal cells in neuroblastic tumors. [Mol Cancer Res.](http://www.ncbi.nlm.nih.gov/pubmed/21642390) 2011;(7):815-23.

E13. [Livak KJ](http://www.ncbi.nlm.nih.gov/pubmed?term="Livak KJ"%5BAuthor%5D) and [Schmittgen](http://www.ncbi.nlm.nih.gov/pubmed?term="Schmittgen TD"%5BAuthor%5D) TD. Analysis of relative gene expression data using real-time quantitative PCR and the 2(-Delta Delta C(T)) Method. [Methods.](http://www.ncbi.nlm.nih.gov/pubmed/11846609) 2001;(4):402-8.

E14. Amrani Y, Syed F, Huang C, Li C, Liu V, Jain D, et al. [Expression and activation of the oxytocin receptor in airway smooth muscle cells: Regulation by TNF alpha and IL-13.](http://www.ncbi.nlm.nih.gov/pubmed/20670427) Respir Res. 2010;29(11):104.

E15. Kearley J, Barker JE, [Robinson](http://www.ncbi.nlm.nih.gov/pubmed?term=Robinson DS%5BAuthor%5D&cauthor=true&cauthor_uid=16314435) DS and [Lloyd](http://www.ncbi.nlm.nih.gov/pubmed?term=Lloyd CM%5BAuthor%5D&cauthor=true&cauthor_uid=16314435) CM. Resolution of airway inflammation and hyperreactivity after *in-vivo* transfer of CD4+CD25+ regulatory T cells is interleukin 10 dependent. [J Exp Med.](http://www.ncbi.nlm.nih.gov/pubmed/16314435) 2005;202(11):1539-47.

E16. Kearley J, [Erjefalt](http://www.ncbi.nlm.nih.gov/pubmed?term=Erjefalt JS%5BAuthor%5D&cauthor=true&cauthor_uid=20971830) JS, [Andersson](http://www.ncbi.nlm.nih.gov/pubmed?term=Andersson C%5BAuthor%5D&cauthor=true&cauthor_uid=20971830) C, [Benjamin](http://www.ncbi.nlm.nih.gov/pubmed?term=Benjamin E%5BAuthor%5D&cauthor=true&cauthor_uid=20971830) E, [Jones](http://www.ncbi.nlm.nih.gov/pubmed?term=Jones CP%5BAuthor%5D&cauthor=true&cauthor_uid=20971830) CP, [Robichaud](http://www.ncbi.nlm.nih.gov/pubmed?term=Robichaud A%5BAuthor%5D&cauthor=true&cauthor_uid=20971830) A, et al. IL-9 governs allergen-induced mast cell numbers in the lung and chronic remodelling of the airways. [Am J Respir Crit Care Med.](http://www.ncbi.nlm.nih.gov/pubmed/?term=Kearley+J+2011) 2011;183(7):865-75.
